# Synergistic changes and interrelationships among chemical composition, aroma, and color during the stir-frying of Sichuan pepper (*Zanthoxylum bungeanum* maxim.)

**DOI:** 10.1016/j.fochx.2026.104644

**Published:** 2026-10-16

**Authors:** Kangjie Li, Jing Wang, Xiaofei Zhang, Qiao Zhang, Jing Sun, Chongbo Zhao

**Affiliations:** a College of Pharmacy, Shaanxi University of Chinese Medicine, Xianyang 712046, PR China; b Shaanxi Traditional Chinese Medicine Processing Technology Heritage Base, and Shaanxi Provincial Engineering Technology Research Center for Traditional Chinese Medicine Decoction Pieces, Shaanxi University of Chinese Medicine, Xi'an 712046, China; c Shaanxi Provincial Engineering Research Center for Traditional Chinese Medicine Aromatherapy Industry in Universities, Xianyang 712046, PR China

**Keywords:** Sichuan pepper, Stir-frying process, Aroma, Color, Bioactive components

## Abstract

This study investigated changes in chemical composition, aroma, and color during Sichuan pepper stir-frying (0–10 min, 120 °C), identifying key contributors and a processing window. An integrated strategy combining colorimetry, electronic nose, HS-GC–MS, HPLC–UV, sensory, antioxidant, and molecular docking/dynamics analyses was applied. Color shifted from red to dark brown, with a* peaking at 5 min, while electronic-nose responses were highest at 4–6 min. Among 26 volatile compounds, (+)-limonene showed the highest ROAV (100), whereas eucalyptol declined sharply after 3 min. Molecular docking and dynamics supported stable binding of (+)-limonene to OR1A2. Total flavonoids and sanshools, as well as antioxidant activity, peaked at 5 min, while polyphenols continuously decreased. Multivariate analyses identified seven key contributors (VIP > 1), and correlation analysis showed associations of L* with bioactive constituents and a* with numbing-related constituents and antioxidant activity. 4–5 min was identified as a reference processing window for quality control.

## Highlights

• Stir-frying 4–5 min balanced aroma, color, and bioactives in Sichuan pepper.
• L* value correlated with flavonoids/polyphenols, a visual quality indicator.
• (+)-Limonene (ROAV=100) is a key aroma driver and binds stably to OR1A2.
• Total flavonoids and eucalyptol (VIP>1) drive the aroma–color synergy.
• Eucalyptol declines sharply after 3 min, a potential thermal marker.

## 1 Introduction

Sichuan pepper is the dried mature pericarp of plants belonging to the genus *Zanthoxylum* in the family Rutaceae. Originating in China, it has a long history of cultivation and is widely distributed across Sichuan, Shaanxi, Gansu, Chongqing, and other regions (Gao et al., 2024). As a multifunctional economic crop that serves medicinal, culinary, and seasoning purposes, Sichuan pepper holds a prominent place in Chinese dietary culture, imparting a unique numbing and aromatic flavor to dishes (Ivane et al., 2022; J. Li et al., 2022). The quality of Sichuan pepper used in food processing is predominantly determined by its flavor and color, both of which are closely associated with its chemical composition (K. Zhang et al., 2026). The Chinese Pharmacopoeia stipulates that stir-fried Sichuan pepper should be processed until it develops a characteristic aroma (Chinese Pharmacopoeia Commission, 2025). This characteristic is primarily derived from volatile constituents such as terpenes, aldehydes, and ketones present in the essential oil, whereas the color is related to pigments, including flavonoids and polyphenols, as well as Maillard reaction and caramelization products formed during stir-frying (Qiu et al., 2025; W. Zhang et al., 2025).

Stir-frying is a critical step in Sichuan pepper processing, as it removes the raw, green odor, triggers and enhances the characteristic numbing and aromatic flavor, and simultaneously improves the color (Zhao et al., 2022). Previous studies have shown that stir-frying markedly affects the volatile profile and aroma characteristics of Sichuan pepper. Li et al. (Z. Li et al., 2019) reported that changes in cooking temperature significantly altered aroma-active compounds, particularly terpenoids such as limonene and myrcene. Liu et al. (Liu et al., 2022) further demonstrated that frying temperature and duration affected the volatile composition and sensory characteristics of Sichuan pepper oil, with changes in linalool and other aroma-related compounds contributing to differences in citrus and floral notes. More recently, Gu et al. (Gu et al., 2025) investigated aroma formation during the frying process and showed that heat treatment reduced fresh, floral, and citrus notes while enhancing roasted and fatty notes, and identified several aroma-active compounds associated with these changes. These studies demonstrate that thermal processing substantially reshapes the aroma profile of Sichuan pepper. However, existing studies on Sichuan pepper stir-frying have mainly focused on individual indicators, such as volatile compounds, numbing substances, aroma, or color, whereas systematic investigations into the coordinated changes and interrelationships among chemical composition, aroma, color, and sensory quality during stir-frying remain limited (Dai et al., 2026; Wu et al., 2026).

Chemical composition, aroma, and color, as core quality indicators of Sichuan pepper, do not exist in isolation but are closely interconnected and may change synergistically interrelated (Fei et al., 2025). The volatile compounds in the essential oil not only serve as the material basis of aroma, but their oxidation and decomposition may also affect the color of Sichuan pepper. Flavonoids and polyphenols act both as antioxidant substances and as important pigments that influence color, and they may simultaneously interact with the formation or transformation of volatile constituents (He et al., 2025; Ma et al., 2019; Zhu et al., 2025). However, systematic studies on the synergistic changes and interrelationships among chemical composition, aroma, and color during stir-frying remain limited. Therefore, this study aimed to elucidate their dynamic changes and interrelationships, identify the key components contributing to quality changes, determine an appropriate stir-frying window, and evaluate the potential of L* and a* as visual indicators of quality changes during stir-frying. By integrating chemical, sensory, and instrumental analyses, this study provides a more comprehensive understanding of quality transformations in Sichuan pepper during stir-frying.

## 2 Materials and methods

### 2.1 Materials

Sichuan pepper (the dried mature pericarp of *Zanthoxylum bungeanum* Maxim., Rutaceae) was purchased from Shaanxi Xingshengde Pharmaceutical Co., Ltd. (Lot No. 20251201; Hancheng, Shaanxi, China) and authenticated by Professor Changli Wang at Shaanxi University of Chinese Medicine.

1,2-Dichlorobenzene (≥ 99.9%, W13876), Folin–Ciocalteu reagent, and DPPH (2,2-diphenyl-1-picrylhydrazyl, 98%, V93833) were obtained from Shanghai Yuanye Bio-Technology Co., Ltd. Rutin (≥ 98.0%, PS1930–0100) was supplied by Chengdu Push Bio-Technology Co., Ltd. Hydroxy-α-sanshool (≥ 98.0%, STC5640120), hydroxy-β-sanshool (≥ 98.0%, STC2010110), and xanthoxylin (≥ 98.0%, ST79640120) were purchased from Shanghai Shidande Standard Technical Service Co., Ltd. All other reagents were of analytical grade and obtained from Sinopharm Chemical Reagent Co., Ltd.

### 2.2 Preparation of samples with different stir-frying durations

Following the plain stir-frying procedure specified in the General Principles of Processing (0213) of the Chinese Pharmacopoeia (2025 Edition), a 26 cm stainless-steel wok was placed on an electric ceramic cooktop with a heating power of 2000 W and preheated until the temperature at the center of the wok bottom reached 120 °C, as monitored by an infrared thermometer. The temperature of 120 °C was selected as a controllable temperature within the reported range for low-heat stir-frying of Sichuan pepper and was maintained using the electric ceramic cooktop. For each stir-frying time point, three independent 50 g batches of Sichuan pepper were prepared separately. Each batch was processed independently under the same stir-frying conditions. For assays involving repeated instrumental measurements, the measurements within each independent batch were averaged to obtain one representative value for that batch. The three independent batch values were then used for statistical analysis. Based on preliminary trials showing distinct changes in color, aroma, and chemical composition during the early and middle stages of stir-frying, a 1 min sampling interval was selected to capture the critical changes around the 5 min stage. For comparison, 50 g of Sichuan pepper was stir-fried in a conventional iron wok over an open flame following the traditional empirical endpoint of “stir-fried until fragrant.” After removal, the samples were naturally cooled to room temperature, ground using a grinder, passed through a 40-mesh sieve, sealed in light-proof bags, and stored at 4 °C until analysis.

### 2.3 Determination of color values of samples with different stir-frying durations

With reference to a previous study (Wang et al., 2026) and with slight modifications, a CS-520 spectrophotometer (Hangzhou CHNSpec Technology Co., Ltd., China) was calibrated with a standard white tile. Accurately weighed 5 g of Sichuan pepper powder was placed in a petri dish and spread evenly. Three measuring points at different positions were selected, and the L*, a*, and b* values were recorded; the average values were calculated. The color difference ΔE was computed using the following formula:

$$\mathrm{\Delta E}=\sqrt{{\left({\mathrm{\Delta L}}^{*}\right)}^{2}+{\left({\mathrm{\Delta a}}^{*}\right)}^{2}+{\left({\mathrm{\Delta b}}^{*}\right)}^{2}}$$

Where ΔL* = L*_1_ – L*_0_,Δa* = a*_1_ - a*_0_,Δb* = b*_1_ - b*_0_; L*_0_, a*_0_, b*_0_ denote the color parameters of the unstir-fried Sichuan pepper (0 min), whereas L*₁, a₁, and b₁ denote those of the samples at different stir-frying stages.

### 2.4 Scanning Electron microscopy (SEM) observation of surface morphology

Intact Sichuan pepper pericarps were cut into small pieces, mounted onto a specimen stub using conductive adhesive, and sputter-coated with gold (15 nm). The surface morphology was observed at 500× magnification using a VEGA3 scanning electron microscope (SEM, TESCAN Trading (Shanghai) Co., Ltd., Shanghai, China) operated at an accelerating voltage of 5 kV. Additional higher-magnification images (2000×) were obtained to provide detailed information on surface micropore formation.

### 2.5 *E*-nose analysis

With reference to a previous method (Qin et al., 2026) and slight modifications, accurately weighed 1 g of Sichuan pepper powder was accurately weighed, placed into a 20 mL headspace vial, sealed, and equilibrated in a thermostatic water bath at 45 °C for 20 min. Analysis was performed using an iSensortalk N1400 electronic nose (Beijing iSensortech Technology Co., Ltd., Beijing, China) under the following conditions: injection time, 5 s; purge time, 60 s; acquisition time, 120 s; and pump duty cycle, 20%. Each sample was analyzed in triplicate, and the mean value of the three technical replicates was used for subsequent analysis.

### 2.6 GC–MS determination of volatile compounds in samples with different stir-frying durations

The volatile compounds of Sichuan pepper were analyzed using headspace sampling coupled with a QP2020 gas chromatograph–mass spectrometer (GC–MS, Shimadzu Corporation, Kyoto, Japan), based on a previously reported procedure (Feng et al., 2024) with minor modifications. For sample preparation, 2 g of powder was mixed with 10 μL of 1,2-dichlorobenzene internal standard solution (10 mg/mL) in a headspace vial and sealed. The headspace parameters were as follows: vial equilibration temperature, 80 °C for 30 min, loop temperature, 140 °C; loop equilibration time, 2 min; and injection time, 3 min. GC separation was carried out on a DB-5MS capillary column (30 m × 0.25 mm × 0.25 μm) with helium as the carrier gas at a flow rate of 1.0 mL/min. The oven temperature program was as follows: 50 °C held for 1 min, then increased to 220 °C at 30 °C/min and held for 1 min, followed by an increase to 280 °C at 5 °C/min and held for 5 min. A 1 μL aliquot was injected with a split ratio of 30:1. Mass spectrometric detection was performed using an electron ionization (EI) source at 70 eV with a solvent delay of 3 min. Qualitative identification of volatile compounds was achieved by matching the acquired mass spectra against those in the NIST 17 library. Their semi-quantitative contents were estimated using 1,2-dichlorobenzene as a single internal standard, assuming a response factor of 1.0 for all compounds. Therefore, the calculated values represent approximate contents expressed as 1,2-dichlorobenzene equivalents rather than absolute concentrations.

### 2.7 Relative Odor activity value (ROAV)

The aroma contribution of volatile compounds was evaluated using the relative odor activity value (ROAV) method. The compound that contributed the most to the overall aroma was assigned an ROAV of 100, and the ROAVs of the other constituents were calculated using the following equation: ROAV_i_ ≈ 100 × (C_i_/C_max_) × (T_max_/T_i_), where C_i_ and T_i_ denote the relative content and odor threshold of compound i, respectively, and C_max_ and T_max_ represent the corresponding values for the most aroma-contributing compound. Because the volatile contents (C_i_) were obtained by semi-quantitative analysis using a single internal standard, the calculated ROAVs should be relative estimates of odor contribution rather than absolute odor activity values. Volatile compounds with ROAV ≥ 1 were considered key aroma components contributing substantially to the overall odor profile, while those with 0.1 ≤ ROAV < 1 were regarded as auxiliary aroma compounds that may contribute to odor modification.

### 2.8 Sensory evaluation

Eight professionally trained panelists with relevant expertise participated in the sensory evaluation. The assessment was conducted with reference to the Chinese National Standards GB/T 10220–2012 and GB/T 30391–2013. Samples from different stir-frying times were randomly coded and presented in a randomized order for blinded evaluation. Each sample was evaluated three times, and the mean scores were used for subsequent analysis. Sensory attributes included color, aroma, taste, and burnt odor, with maximum scores of 20, 30, 30, and 20 points, respectively, giving a total sensory score of 100 points. Burnt odor was scored inversely, with higher scores indicating a weaker burnt odor.

The intensity of four sensory attributes, including color, aroma, taste, and burnt odor, was evaluated according to the corresponding criteria and scoring ranges specified in Table 1. Samples were labelled with random codes and presented in a randomized order to minimize bias. Each sample was evaluated three times, and the mean values were used for subsequent analysis.

**Table 1:** GC–MS detection results of Sichuan pepper at different stir-frying times.

RT/min	Name	Types	CAS	Estimated content (mg/kg, as 1,2-dichlorobenzene equivalents)										
				0 min	1 min	2 min	3 min	4 min	5 min	6 min	7 min	8 min	9 min	10 min
5.26	Alpha-Thujene	Monoterpene	2867-5-2	2340.20	504.67	2498.91	546.10	2104.34	2622.98	656.73	2657.16	2569.59	551.77	567.45
5.39	Alpha-Pinene	Monoterpene	80–56-8	3554.22	817.91	4248.21	969.64	3651.49	4548.73	1113.17	4557.01	4395.47	925.36	975.67
5.63	Camphene	Monoterpene	79–92-5	51.97	12.86	67.98	17.65	57.67	77.70	19.76	97.87	92.49	19.46	17.91
6.02	Sabinene	Monoterpene	3387-41-5	10,883.05	2591.97	13,497.17	3019.46	11,512.56	13,885.35	3399.54	13,842.19	13,348.40	2810.71	3056.54
6.09	beta-Pinene	Monoterpene	127–91-3	1335.11	341.97	1788.06	409.19	1505.85	1907.07	479.00	1962.19	1849.75	391.02	409.12
6.23	Myrcene	Monoterpene	123–35-3	4363.01	1077.70	5591.97	1286.07	4774.63	5824.38	1449.07	6009.99	5723.94	1234.20	1350.88
6.48	Alpha-Phellandrene	Monoterpene	99–83-2	984.30	216.96	1181.09	274.25	1006.42	1305.86	336.50	1379.62	1348.41	291.54	314.08
6.67	(+)-2-Carene	Monoterpene	4497-92-1	2861.54	350.72	1606.50	340.09	1337.53	1673.62	427.38	1779.65	1720.78	379.20	410.82
6.8	P-Cymene	Aromatic hydrocarbon	99–87-6	721.70	162.22	818.72	186.73	660.43	848.17	216.48	887.25	866.96	190.79	207.52
6.9	(+)-Limonene	Monoterpene	5989-27-5	10,057.59	2193.19	11,519.27	2625.68	14,292.45	17,198.15	2893.74	17,242.57	16,562.80	2513.40	2763.13
6.94	Eucalyptol	Ether / Oxide	470–82-6	4939.51	1119.02	5509.74	1112.28	1179.21	1269.76	1295.83	1072.98	1046.10	1048.72	1122.99
7.02	Ocimene	Monoterpene	13,877–91-3	50.00	50.00	50.00	50.00	50.00	50.00	50.00	50.00	50.00	50.00	50.00
7.12	Gamma -Terpinene	Monoterpene	99–85-4	1076.33	277.82	1434.62	340.74	1164.21	1496.11	385.73	1620.73	1524.73	328.91	355.38
7.34	4-Thujanol	Alcohol	15,537–55-0	3941.64	513.49	2384.38	510.30	1992.25	2505.99	642.24	2663.61	2629.86	590.16	626.99
7.48	Terpinolene	Monoterpene	586–62-9	1413.50	388.79	1681.47	304.34	1379.68	1402.95	315.65	1102.48	1041.51	188.07	141.96
7.81	Linalool	Alcohol	78–70-6	1363.27	249.88	1203.62	271.07	976.21	1267.54	328.68	1374.56	1332.39	294.21	322.49
7.94	beta-TERPINEOL	Alcohol	7299-40-3	1563.30	275.54	1138.97	210.73	1000.48	1066.28	276.50	1001.70	959.39	205.27	199.35
9.21	Terpinen-4-ol	Alcohol	562–74-3	993.89	121.10	519.48	95.94	468.19	493.73	120.63	451.40	446.49	91.51	98.77
9.41	(+)-alpha-Terpineol	Alcohol	7785-53-7	391.36	59.07	294.27	43.23	283.67	279.89	68.97	252.45	241.27	39.26	49.53
9.84	Sylvestrene	Monoterpene	1461-27-4	48.07	21.03	93.13	19.05	70.10	83.65	21.48	80.74	74.02	13.70	14.85
10.27	Linalyl acetate	Ester	115–95-7	306.15	88.81	407.56	72.54	310.03	317.79	72.30	265.39	237.26	42.28	41.58
10.36	Piperitone	Ketone	89–81-6	505.77	94.85	520.69	94.09	458.27	457.83	114.95	445.73	410.40	78.65	92.02
11.51	Alpha-Terpineol	Alcohol	98–55-5	53.73	12.90	60.98	13.39	63.04	61.62	14.15	38.72	47.23	6.02	8.74
11.68	Alpha-Terpinyl	Ester	80–26-2	289.59	52.78	303.46	54.22	254.00	239.59	58.74	222.64	220.61	42.99	43.74
13.07	Beta-Caryophyllen	Sesquiterpene	87–44-5	144.45	31.10	165.60	29.39	131.26	133.26	32.71	122.69	119.81	23.39	22.73
14.06	Germacrene D	Sesquiterpene	23,986–74-5	72.50	16.28	97.59	18.08	82.85	78.92	18.88	68.43	66.85	12.04	10.26

### 2.9 Molecular docking and molecular dynamics simulation

The three-dimensional structures of core olfactory receptor proteins involved in aroma perception were retrieved from the AlphaFold3 database (Abramson et al., 2024), and the molecular structures of characteristic volatile compounds were obtained from the PubChem database. Seven key aroma compounds were selected based on their ROAV values (ROAV ≥1), relatively high semi-quantitative contents in the volatile profile, and their reported olfactory importance in Sichuan pepper or related spices. These compounds included (+)-limonene, β-terpineol, linalool, eucalyptol, myrcene, and two additional representative aroma compounds. Seven olfactory receptors were selected based on previous reports of interactions between terpenoid compounds and olfactory receptors. All molecular structures were converted to PDB format using Open Babel. Molecular docking was performed with AutoDock Tools, and the binding poses were generated by AutoDock Vina and subsequently visualized. The docking analysis was conducted to provide a molecular-level interpretation of the potential interactions between key aroma compounds and olfactory receptors.

Molecular dynamics (MD) simulation of the complex formed by (+)-limonene and the olfactory receptor OR1A2 was carried out using GROMACS 2020.3 (Abraham et al., 2015). MD simulation was further performed to evaluate the stability of the ligand–receptor complex over time and to provide additional structural evidence for the docking results. The simulation box was sized so that the distance between the protein atoms and the box edges exceeded 1.0 nm, and the system energy was minimized to eliminate steric clashes or atomic overlap. Equilibration was conducted under the NVT and NPT ensembles, followed by a 100 ns production run. Temperature and pressure were controlled using the V-rescale thermostat and the Parrinello–Rahman barostat, respectively. Van der Waals interactions were described by the Lennard-Jones potential with a non-bonded cutoff of 1.4 nm. All bond lengths were constrained using the LINCS algorithm. Electrostatic interactions were treated using the particle-mesh Ewald (PME) method with a Fourier spacing of 0.16 nm.

### 2.10 HPLC–UV determination of non-volatile constituents

Accurately weighed Sichuan pepper powder (0.5 g) was ultrasonically extracted with 25 mL of 60% (*v*/v) ethanol for 30 min and then filtered to obtain the test solution. Reference stock solutions were prepared by individually dissolving accurately weighed amounts of 5-hydroxymethylfurfural (5-HMF), gallic acid, rutin, hydroxy-β-sanshool, hydroxy-α-sanshool, and xanthoxylin in 70% (*v*/v) methanol to yield concentrations of 1.64, 2.18, 1.98, 0.71, 1.98, and 1.87 mg/mL, respectively.

Analysis was performed on an LC-2030 Plus high-performance liquid chromatograph (HPLC, Shimadzu Corporation, Kyoto, Japan) equipped with an ENorMicro ENM-Miracle™ XL-C18 column (250 mm × 4.6 mm, 5 μm). The mobile phase consisted of methanol (A) and water (B) with the following gradient program: 0–15 min, 32% A; 15–25 min, 32–55% A; 25–35 min, 55–58% A; 35–52 min, 58% A. The flow rate was 1.0 mL/min, the column temperature was maintained at 30 °C, and the detection wavelength was set at 270 nm. The method was validated by assessing precision, stability, repeatability, and recovery, and the relative standard deviations (RSDs) were calculated to evaluate the reliability of the analytical method. The linear regression equations, linearity ranges, and correlation coefficients are presented in Table S2, while the results of the precision, stability, repeatability, and recovery tests (*n* = 6) are summarized in Table S3.

### 2.11 Determination of Total flavonoids

A series of rutin standard solutions (0, 0.5, 1.0, 1.5, 2.0, 2.5, and 3.0 mL of 0.2 mg/mL rutin) were prepared accurately. The absorbance was measured at 510 nm using the NaNO_2_–Al(NO_3_)_3_ colorimetric method. A linear regression equation was established with rutin concentration (x) as the abscissa and absorbance (y) as the ordinate: y = 2.21× + 0.0469 (R^2^ = 0.9998). The absorbance of the Sichuan pepper test solution was determined under the same conditions, and the total flavonoid content was calculated using the regression equation.

### 2.12 Determination of Total polyphenols

Gallic acid (10 mg) was accurately weighed, dissolved in water, and diluted to 10 mL to obtain a 1 mg/mL standard stock solution. Aliquots of 0.2, 0.4, 0.6, 0.8, and 1.0 mL were transferred to 10 mL volumetric flasks, and 1 mL of 1 mol/L Folin–Ciocalteu reagent and 2 mL of 15% (*w*/*v*) sodium carbonate solution were added sequentially. The mixtures were diluted to volume with water, mixed thoroughly, and allowed to stand for 1 h. The absorbance was measured at 765 nm against a blank. A linear regression equation was obtained with gallic acid concentration (x) as the independent variable and absorbance (y) as the dependent variable: y = 1.939× + 0.2626 (R^2^ = 0.9998). One milliliter of the Sichuan pepper test solution was subjected to the same procedure, and the total polyphenol content was calculated using the standard curve.

### 2.13 Antioxidant activity assays

**DPPH radical scavenging assay:** The sample solution was mixed with an equal volume of 0.2 mmol/L DPPH solution and incubated in the dark for 30 min. The absorbance was measured at 517 nm (A₁). A blank control (A₀) using 60% ethanol and a sample background control (A₂) were prepared concurrently. The scavenging rate was calculated as: scavenging rate (%) = [1 − (A₁ − A₂) / A₀] × 100%.

**ABTS radical cation scavenging assay:** Ten microliters of sample, Trolox standard, or water were added to the wells of a microplate, followed by the sequential addition of 20 μL peroxidase and 170 μL ABTS working solution. After 6 min reaction, the absorbance was recorded at 414 nm. Results were expressed as Trolox equivalent antioxidant capacity (TEAC) in mmol TE/g DW.

**Superoxide anion radical scavenging assay:** Buffer solution and the respective solutions were added to the measurement tube (sample), control tube (water), and standard tube (0.15 mg/mL ascorbic acid). Reagents 2, 3, and 4 were then added in sequence, and the mixtures were incubated at 37 °C for 40 min. After incubation, the chromogenic agent was added and the tubes were left to stand for 10 min before measuring the absorbance was measured at 550 nm. The activity was calculated as: activity (U/L) = (OD_control − OD_sample) / (OD_control − OD_standard) × 0.15 × 1000 × dilution factor.

**Hydroxyl radical (·OH) scavenging assay:** Fifty microliters each of FeSO₄ solution, H₂O₂ solution, salicylic acid–ethanol solution, and the sample were combined, and the volume was made up to 400 μL. After incubation at 37 °C for 30 min, the absorbance was measured at 510 nm (A_sample). A blank (A_blank) and a sample background (A_background) were measured in parallel. The scavenging rate was calculated as: scavenging rate (%) = [1 − (A_sample − A_background) / A_blank] × 100%.

### 2.14 Statistical and correlation analysis

All results are expressed as mean ± standard deviation. Statistical analyses were performed using R software (version 4.5.1). Comparisons among different stir-frying durations were conducted by one-way analysis of variance (ANOVA) followed by Tukey's honestly significant difference (HSD) post hoc test, with *P* < 0.05 considered statistically significant. Pairwise correlations were assessed using Spearman's rank correlation coefficients and a correlation heatmap was generated (significance thresholds: *P* < 0.05, *P* < 0.01, and *P* < 0.001). Furthermore, partial least squares regression (PLSR) was employed to model the relationships between chemical constituents and sensory attributes (aroma and color), and the variable importance in projection (VIP) values were used to identify key contributing components.

## 3 Results and discussion

### 3.1 Changes in appearance and powder morphology of Sichuan pepper during stir-frying

With the extension of stir-frying time, the color of Sichuan pepper pericarp gradually deepened from the initial bright red (Fig. 1A). A glossy, oily sheen appeared on the surface at 1–3 min of stir-frying. After 4–6 min, the pericarp color turned reddish-brown, and partial charring appeared at the edges of some pericarps from 7 min onward. At 10 min, the samples were uniformly dark brown and emitted a burnt odor. The temperature–time profiles of the electric ceramic cooktop and traditional open-flame stir-frying are shown in Fig. S1. Compared with the open-flame method, the electric ceramic cooktop exhibited a more stable temperature profile with smaller fluctuations during stir-frying, indicating better temperature controllability and process stability. The color change trend of the powder was consistent with that of the intact pericarp: the unstir-fried sample powder was light reddish-yellow, whereas the powder became dark reddish-brown in the later stages of stir-frying. SEM (Fig. 1B) observations revealed that the surface of unstir-fried Sichuan pepper was relatively smooth with an intact waxy layer. After 3 min of stir-frying, the waxy layer started to melt and rupture, micropores appeared on the surface, and a small amount of oil was observed around the sample, corresponding to the “sweating” phenomenon observed during stir-frying. At 5 min, the surface structure visibly collapsed and the number of pores increased, which may have facilitated the release and diffusion of volatile compounds. The higher-magnification SEM images (2000×, Fig. S2) further showed the formation of surface micropores, localized oil release, and progressive tissue collapse, providing additional microscopic evidence for the structural changes observed during stir-frying. With prolonged heating, excessive tissue collapse and charring were observed, providing structural evidence of progressive tissue deterioration at later stages. At 8 min, charred fragments appeared on the surface, indicating severe tissue damage. These findings suggest that moderate stir-frying disrupts the waxy barrier on the pericarp surface and may facilitate the release of volatile compounds, providing a structural basis for the characteristic requirement that Sichuan pepper be “stir-fried until fragrant.” However, excessive stir-frying leads to pronounced tissue deterioration, accompanied by aroma loss and the generation of burnt off-odors, underscoring the importance of controlling the degree of stir-frying for quality preservation (Alim et al., 2025; Yang & Jin, 2024). The observed progressive disruption of the surface structure is consistent with previous reports that thermal processing can alter plant tissue structure and enhance volatile release (Zuo et al., 2023).

**Fig. 1:**
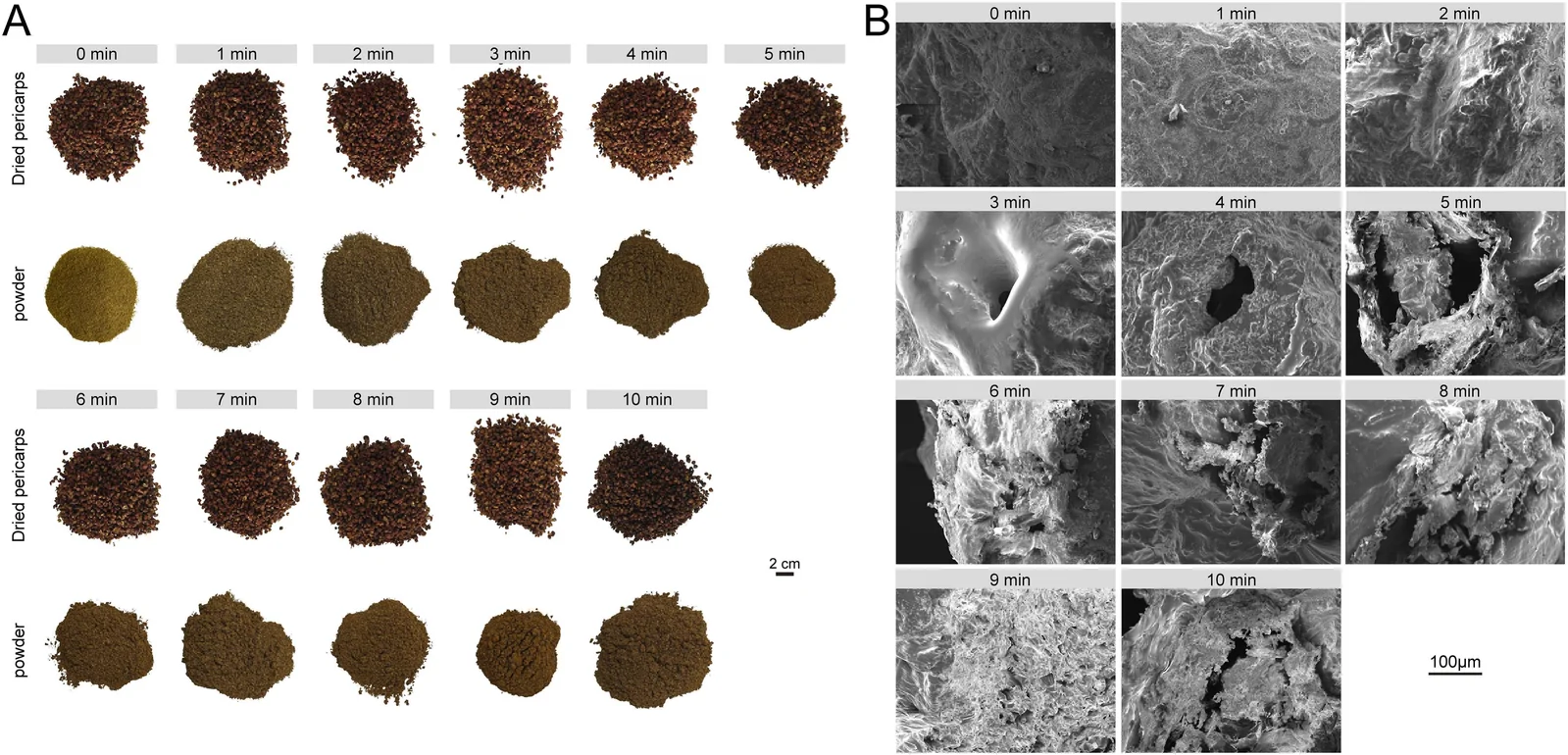
Changes in appearance and surface morphology of Sichuan pepper during stir-frying. (A) Macroscopic appearance of intact pericarps and powder at different stir-frying times (0–10 min). (B) Scanning electron microscopy (SEM) images (500× magnification) showing the evolution of the waxy layer, pore formation, and tissue collapse.

### 3.2 Changes in color values

The changes in the color values of Sichuan pepper powder with stir-frying time are illustrated in Fig. 2A-D. The L* value declined continuously, the a* value increased initially, peaked at 5 min, and then decreased, whereas the b* value decreased throughout the process. The total color difference ΔE exhibited a corresponding trend. During 4–6 min of stir-frying, the color shifted to a deeper reddish-brown, and by 10 min, it approached a dark, burnt appearance. The persistent decrease in L* and the turning point in a* indicated that browning reactions intensified markedly after 5 min, which may have been primarily associated with the accumulation of Maillard reaction and caramelization products as well as the oxidative polymerization of phenolic compounds (Khan et al., 2020). The a* value can serve as a sensitive indicator of the loss of the characteristic “reddish” hue (Song et al., 2025). The rapid decline in a* after 5 min marked a critical turning point at which browning reactions accelerate, thus providing a useful reference for color control. The increase in a* during the early stage suggests that moderate heating favored the retention or exposure of red pigments before extensive browning occurred. After 5 min, the subsequent decrease in a* together with the continuous decline in L* indicated that pigment transformation and browning reactions became increasingly dominant. Cluster analysis and the PCA score plot (Fig. 2E, F) further classified the samples into three distinct groups: 0–2 min, 3–6 min, and 7–10 min, corresponding to the under-stir-fried, moderately stir-fried, and over-stir-fried stages, respectively.

**Fig. 2:**
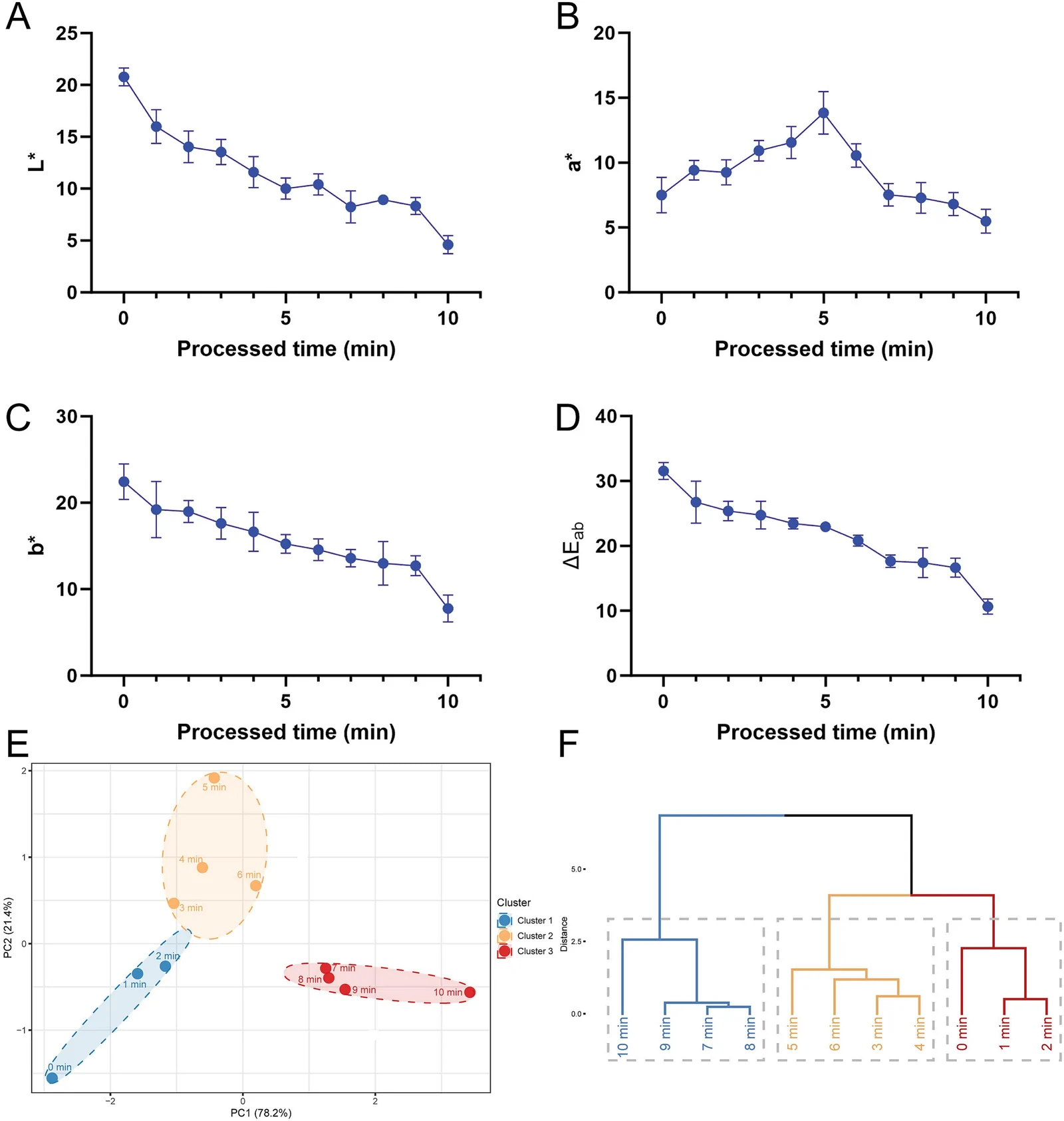
Colorimetric changes of Sichuan pepper powder during stir-frying. (A) L* value (lightness). (B) a* value (red-green). (C) b* value (yellow-blue). (D) Total color difference (ΔE) relative to the unstir-fried sample. (E) Principal component analysis (PCA) score plot of samples categorized by stir-frying time. (F) Hierarchical cluster analysis dendrogram based on color parameters. (For interpretation of the references to color in this figure legend, the reader is referred to the web version of this article.)

### 3.3 *E*-nose response characteristics

The electronic nose sensor responses are presented in Fig. 3A. The unstir-fried sample elicited weak responses, predominantly from sensor S6 (sensitive to alcohols, ethers, and acids) and S10 (sensitive to nitrogen-containing compounds), indicating a relatively weak volatile profile and a subdued aroma in the raw material. After 1–3 min of stir-frying, the responses of sensors targeting aromatics and nitrogen oxides increased. Between 4 and 6 min, the sensor responses reached their maximum, particularly for S6 and S10, indicating the strongest overall sensor response during the middle stage of processing. After 7 min, the responses began to decline, consistent with progressive loss or transformation of volatile compounds. The enhanced response during 4–6 min may reflect the combined effects of matrix disruption and thermal volatilization. After 7 min, the declining response suggests that volatile loss and thermal transformation increasingly outweighed the release of volatile compounds. Cluster analysis and PCA (Fig. 3B, C) showed that the samples were regularly distributed along PC1 (78.2% contribution rate), with the 0–2 min samples distinctly separated from those collected at 3–10 min. Furthermore, the 5 min and 7 min samples were also clearly discriminated, confirming the strong effect of stir-frying time on the measured aroma-related sensor responses (Ding et al., 2025).

**Fig. 3:**
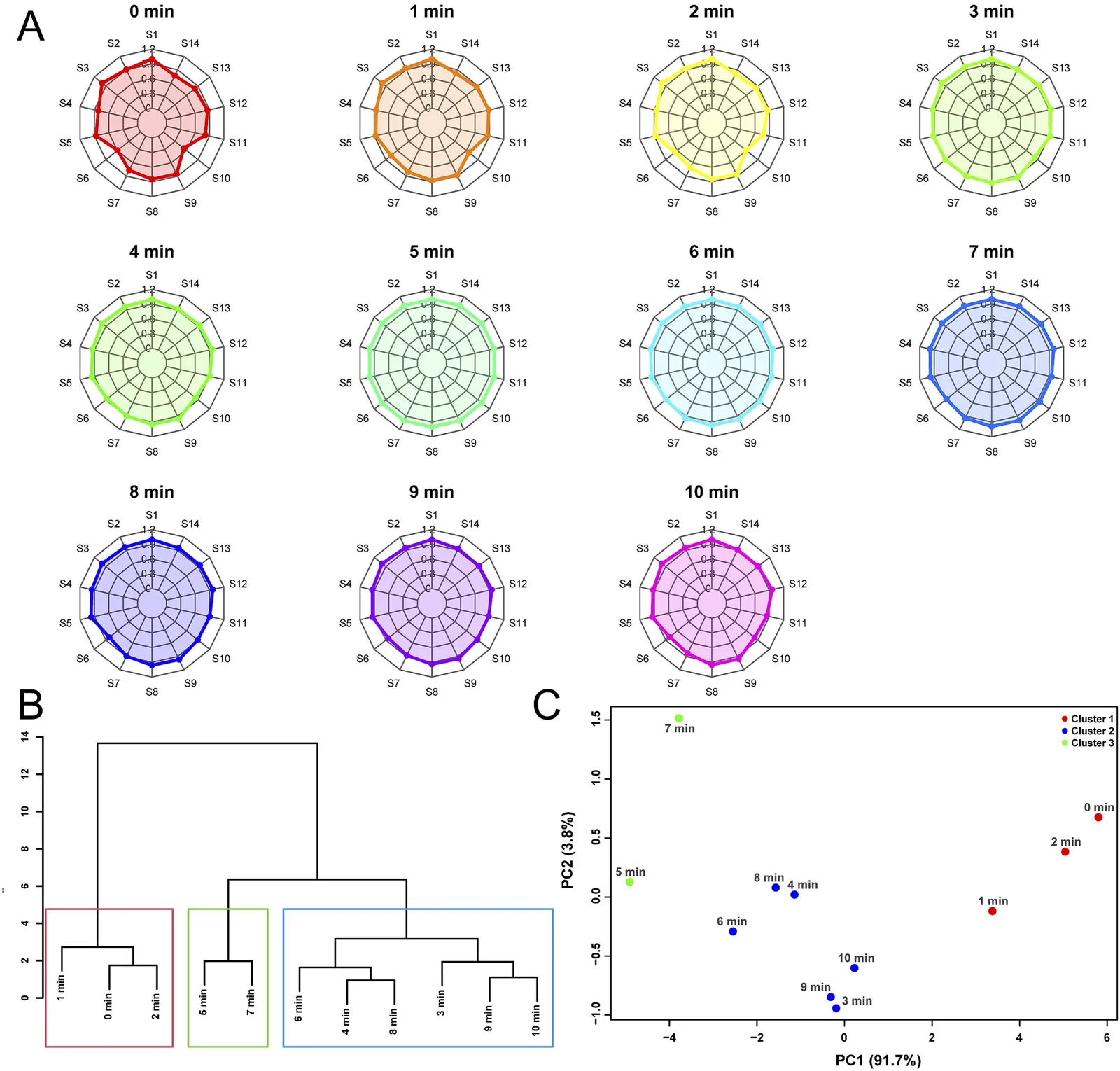
Electronic nose response characteristics of Sichuan pepper at different stir-frying stages. (A) Radar chart of sensor responses. (B) Hierarchical cluster analysis dendrogram. (C) PCA score plot based on electronic nose data.

### 3.4 Sensory evaluation of Sichuan pepper during stir-frying

Sensory evaluation further characterized the quality changes during stir-frying. The sensory profiles are shown in Fig. 4A. Aroma and taste scores generally increased during the early-to-middle stages, whereas prolonged heating resulted in decreased aroma and color scores and a marked decline in the burnt-odor score, indicating a stronger perception of burnt odor. The sensory profiles of the samples were further discriminated by hierarchical cluster analysis (HCA) and principal component analysis (PCA) (Fig. 4B, C). HCA separated the samples into three groups, namely 0–7 min, 8–9 min, and 10 min, with the 10 min sample forming a distinct cluster. PCA showed that PC1 and PC2 explained 91.8% and 7.4% of the total variance, respectively, accounting for 99.2% of the cumulative variance. These results demonstrate that prolonged heating markedly altered the sensory profile. Overall, the sensory evaluation was consistent with the instrumental analyses and indicated that approximately 4–6 min represented an important transition stage associated with favorable aroma and taste characteristics before the development of pronounced burnt odor.

**Fig. 4:**
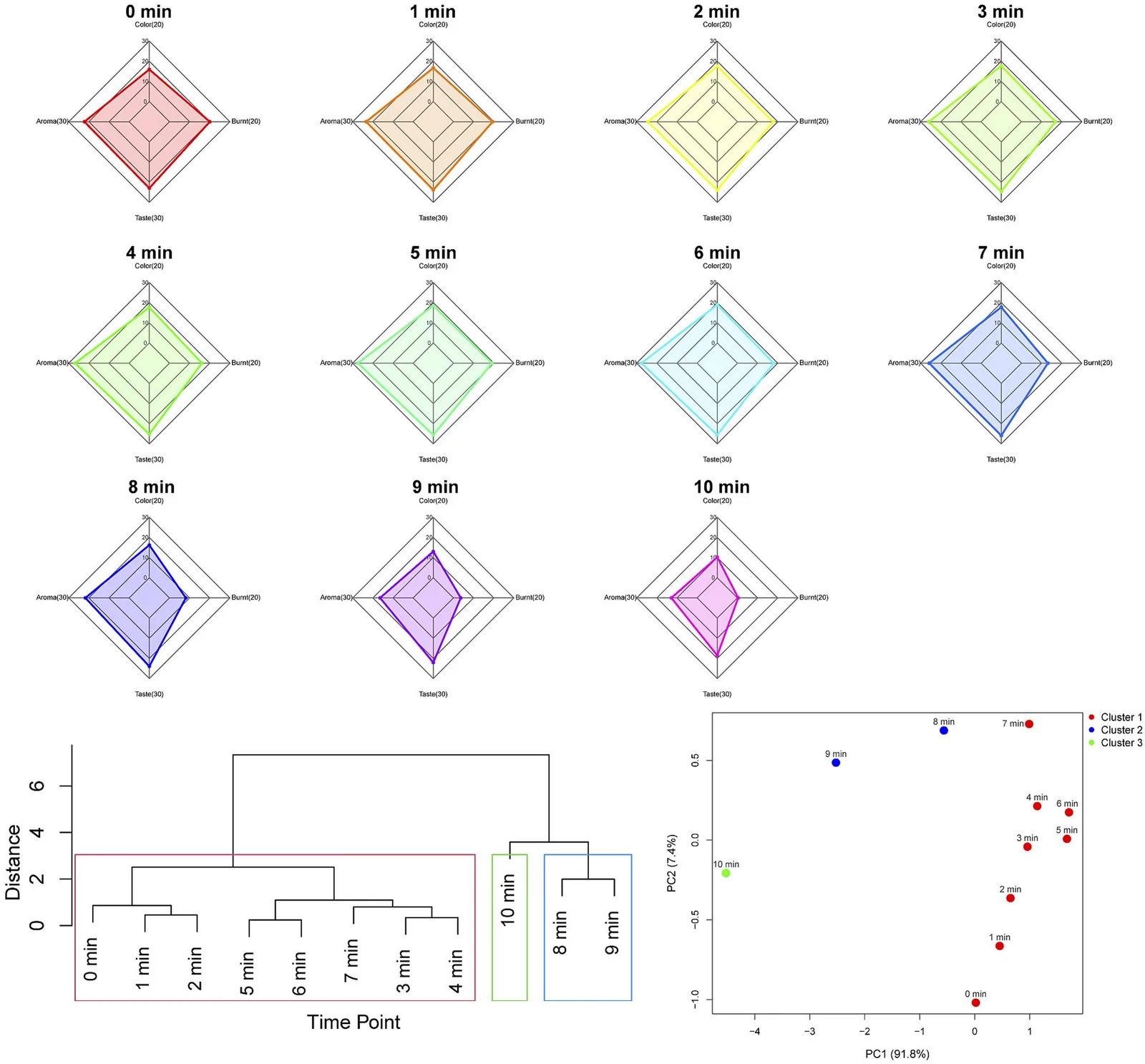
Sensory evaluation and multivariate analysis of Sichuan pepper during stir-frying. (A) Radar plots of sensory profiles at different stir-frying times, including color, aroma, taste, and burnt odor. (B) Hierarchical cluster analysis based on sensory attributes. (C) PCA score plot of samples based on sensory evaluation.

### 3.5 GC–MS analysis of volatile compounds

A total of 26 volatile compounds were identified by headspace GC–MS (Table 1), including monoterpenes, alcohols, esters, sesquiterpenes, ethers/oxides, and ketones. In the raw sample, sabinene, (+)-limonene, eucalyptol, myrcene, and 4-thujanol were the most abundant constituents, with monoterpenes dominating the volatile profile. During stir-frying, the majority of monoterpenes exhibited a pattern of initially increasing and subsequently decreasing. (+)-limonene, α-pinene, sabinene, and myrcene increased slightly within 0–2 min, generally declined at 3 min, increased again to peak levels at 4–5 min, and then declined progressively after 6 min. In the early stage of stir-frying, the melting and rupture of the waxy layer and the disruption of cellular structure may have promoted the desorption and release of monoterpenes from the matrix, leading to a transient increase in their content. This initial increase followed by a decline is consistent with previous reports that thermal treatment can first promote the release of aroma-active terpenoids from plant matrices and subsequently accelerate their evaporation and degradation (Z. Li et al., 2019; Mahanta et al., 2021). Eucalyptol, by contrast, exhibited a different pattern: its content dropped sharply after 3 min and remained at a low level thereafter, indicating relatively high thermal sensitivity. Although the boiling point of eucalyptol (174–177 °C) is similar to that of (+)-limonene (176 °C), its more pronounced decrease may be related to the greater susceptibility of its ether/oxide structure to thermal oxidation. Previous studies have confirmed that high-temperature heating can accelerate the oxidation of Sichuan pepper essential oil and cause flavor deterioration (Chen et al., 2024). The distinct behavior of eucalyptol further indicates that individual volatile compounds differ in thermal stability, making its sharp decline after 3 min particularly informative for monitoring thermal stress during stir-frying. Therefore, eucalyptol can be regarded as a potential chemical marker for monitoring the degree of stir-frying. Among the alcohols, linalool decreased continuously, and 4-thujanol showed a decline of 84.1%. Both linalool and 4-thujanol belong to the monoterpene alcohols, and their persistent decrease is attributable not only to thermal evaporation and oxidative losses but also potentially to acid-catalyzed hydration/dehydration and rearrangement reactions as important degradation pathway. Using isotope tracer techniques, Dong et al. systematically summarized the degradation pathways of linalool and limonene under boiling conditions, confirming that changes in the overall content of terpenoids directly affect the overall aroma characteristics of Sichuan pepper aqueous extracts (Dong et al., 2024).

### 3.6 ROAV analysis of key aroma components

The ROAV values were calculated according to the method described in the literature (Su et al., 2025), with ROAV ≥ 1 indicating key aroma components and 0.1 ≤ ROAV < 1 indicating auxiliary aroma components (Table 2). In the raw Sichuan pepper, 15 key aroma compounds were identified. β-Terpineol showed the highest relative odor contribution (ROAV = 100), followed by (+)-limonene (70.04), linalool (61.69), and eucalyptol (50.85), indicating that these compounds contributed substantially to the volatile profile. During the initial stage of stir-frying, β-terpineol remained the highest-contributing compound at 1 and 2 min (ROAV = 100), but its relative contribution generally decreased thereafter, reaching 71.39 at 5 min and 54.98 at 10 min, although some fluctuations were observed. From 3 min onward, (+)-limonene became the highest relative contributor (ROAV = 100). Linalool decreased from 61.69 in the raw sample to 35.13 at 5 min, followed by fluctuations at later stages. Eucalyptol showed a marked nonlinear change, increasing from 50.85 in the raw sample to 62.62 at 3 min, then decreasing sharply to 12.20 and 10.91 at 4 and 5 min, respectively, followed by a marked increase at 6 min. In contrast, myrcene consistently maintained an ROAV ≥ 1 throughout the process, with ROAV values ranging from 22.46 to 37.01, suggesting a relatively stable contribution to the volatile profile. Importantly, the non-uniform changes in ROAVs indicate that stir-frying altered the relative contribution of individual volatiles rather than simply reducing overall aroma intensity. The decline in heat-sensitive compounds together with the persistence of myrcene therefore reflects a progressive remodeling of the aroma-active profile. Overall, the ROAV results demonstrated progressive remodeling of the aroma-active composition with increasing stir-frying time, characterized by the decline of several heat-sensitive monoterpenes and terpene alcohols and the relative persistence of selected components. The number of key aroma components remained within 15–16 throughout the stir-frying process, while 4–6 compounds were classified as auxiliary modifying components. At approximately 5 min, (+)-limonene remained the dominant relative contributor, while β-terpineol, linalool, myrcene, and other key aroma compounds retained relatively high ROAVs, indicating a distinct transition stage in the aroma-active profile. After 8–10 min, several aroma-active compounds showed further decreases in ROAV, although (+)-limonene and myrcene remained relatively prominent, indicating continued restructuring of the volatile profile rather than a uniform loss of aroma activity.

**Table 2:** Analysis of Odor Activity Values of Volatile Sichuan Pepper Subjected to Different Roasting Durations.

ROAV	Odor threshold mg/kg	Flavor description	ROAV values at different stir-frying times										
			0	1	2	3	4	5	6	7	8	9	10
Alpha-Thujene	22	Woody aroma	0.03	0.02	0.03	0.03	0.02	0.02	0.04	0.02	0.02	0.03	0.03
Alpha-Pinene	0.274	Spicy	3.07	3.23	3.78	4.58	3.17	3.28	4.77	3.28	3.29	4.57	4.38
Camphene	0.015	Pine, earthy	0.82	0.93	1.11	1.52	0.91	1.02	1.55	1.29	1.27	1.76	1.47
Sabinene	0.98	Woody, pine-like	2.63	2.86	3.36	3.99	2.79	2.80	4.08	2.79	2.80	3.88	3.84
beta-Pinene	0.14	Pine-like, resinous	2.26	2.64	3.12	3.78	2.56	2.69	4.02	2.76	2.71	3.78	3.60
Myrcene	0.046	Floral	22.46	25.34	29.65	36.20	24.69	25.03	37.01	25.76	25.54	36.29	36.14
Alpha-Phellandrene	0.04	Black pepper-like, minty	5.83	5.87	7.20	8.88	5.99	6.45	9.88	6.80	6.92	9.86	9.66
(+)-2-Carene	0.21	Turpentine-like	3.23	1.81	1.87	2.10	1.52	1.58	2.39	1.67	1.68	2.44	2.41
P-Cymene	0.0133	Musty, damp rag-like	12.85	13.19	15.01	18.18	11.81	12.61	19.12	13.15	13.38	19.41	19.20
(+)-Limonene	0.034	Citrus, lemon-like	70.04	69.76	82.63	100.00	100.00	100.00	100.00	100.00	100.00	100.00	100.00
Eucalyptol	0.023	Eucalyptus, herbal, camphor-like, medicinal	50.85	52.62	58.43	62.62	12.20	10.91	66.20	9.20	9.34	61.68	60.08
Ocimene	0.034	Herbal, floral, fruity	7.49	8.84	10.29	12.98	8.15	8.70	13.33	9.40	9.21	13.09	12.86
Gamma -Terpinene	1	Pine resin-like, mild woody	0.93	0.56	0.58	0.66	0.47	0.50	0.75	0.53	0.54	0.80	0.77
4-Thujanol	0.03	Balsamic	11.16	14.02	13.67	13.14	10.94	9.25	12.36	7.25	7.13	8.48	5.82
Terpinolene	0.2	Pine resin-like, mild woody	1.61	1.35	1.47	1.76	1.16	1.25	1.93	1.36	1.37	1.99	1.98
Linalool	0.006	Floral, fresh, woody, sweet	61.69	49.67	46.30	45.48	39.67	35.13	54.15	32.92	32.82	46.28	40.88
beta-TERPINEOL	0.003	Fir needle-like, minty	100.00	100.00	100.00	92.75	82.22	71.39	89.87	52.56	56.42	65.23	54.98
Terpinen-4-ol	1.2	Peppery, woody	0.20	0.11	0.11	0.10	0.09	0.08	0.12	0.07	0.08	0.10	0.10
(+)-alpha-Terpineol	0.3	Clove-like	0.31	0.21	0.24	0.19	0.22	0.18	0.27	0.17	0.17	0.18	0.20
Sylvestrene	0.0024	Woody, iris-like floral	4.74	9.48	9.46	10.28	6.95	6.89	10.52	6.63	6.33	7.72	7.61
Linalyl acetate	1	Pleasant aromatic, fruity	0.07	0.10	0.10	0.09	0.07	0.06	0.08	0.05	0.05	0.06	0.05
Piperitone	0.68	Peppery, earthy	0.18	0.15	0.19	0.18	0.16	0.13	0.20	0.13	0.12	0.16	0.17
Alpha-Terpineol	0.23	Clove-like floral	0.06	0.06	0.06	0.08	0.07	0.05	0.07	0.03	0.04	0.04	0.05
Alpha-Terpinyl	2.5	Fresh sweet, lemon-like, lavender-like	0.03	0.02	0.03	0.03	0.02	0.02	0.03	0.02	0.02	0.02	0.02
Beta-Caryophyllen	0.064	Sweet, woody, spicy	0.53	0.53	0.63	0.59	0.49	0.41	0.60	0.38	0.38	0.49	0.44
Germacrene D	1.2	Woody aroma	0.01	0.01	0.02	0.02	0.02	0.01	0.02	0.01	0.01	0.01	0.01
major components			15	15	16	16	15	16	16	16	16	16	16
modifying components			6	6	5	5	5	4	5	4	4	5	5

### 3.7 Molecular docking and dynamics simulation reveal key aroma molecules

Based on ROAV values, semi-quantitative abundance, and reported olfactory importance, seven key aroma compounds were selected for molecular docking with seven olfactory receptors. Molecular docking of the seven key aroma compounds with seven olfactory receptor proteins was performed (Fig. 5A). The binding energies ranged from −4.646 to −7.317 kcal/mol. Notably, the complex between (+)-limonene and OR1A2 exhibited the lowest binding energy of −7.317 kcal/mol, suggesting a relatively favorable binding interaction (< −7.0 kcal/mol). As shown in Fig. 5B, (+)-limonene primarily formed hydrophobic interactions with residues Ala108, Tyr276, and Phe206, while a π–σ interaction was observed between Tyr258 and limonene. Based on this favorable docking result, a 100 ns molecular dynamics simulation was conducted. Root-mean-square deviation (RMSD) and root-mean-square fluctuation (RMSF) analyses (Fig. 5C) revealed that the complex system approached equilibrium after approximately 20 ns, with low RMSD values indicating limited structural fluctuations. The RMSF results (Fig. 5F) showed that the local conformation of the receptor underwent minor adjustments upon binding, whereas the core region maintained consistently low RMSF values, indicating a relatively rigid structural scaffold. The radius of gyration (Rg) fluctuated between 2.08 and 2.20 nm, suggesting that the overall structure of the complex remained relatively compact throughout the simulation. The free energy landscape displayed multiple low-energy clusters, further supporting the conformational stability of the (+)-limonene–OR1A2 complex.

**Fig. 5:**
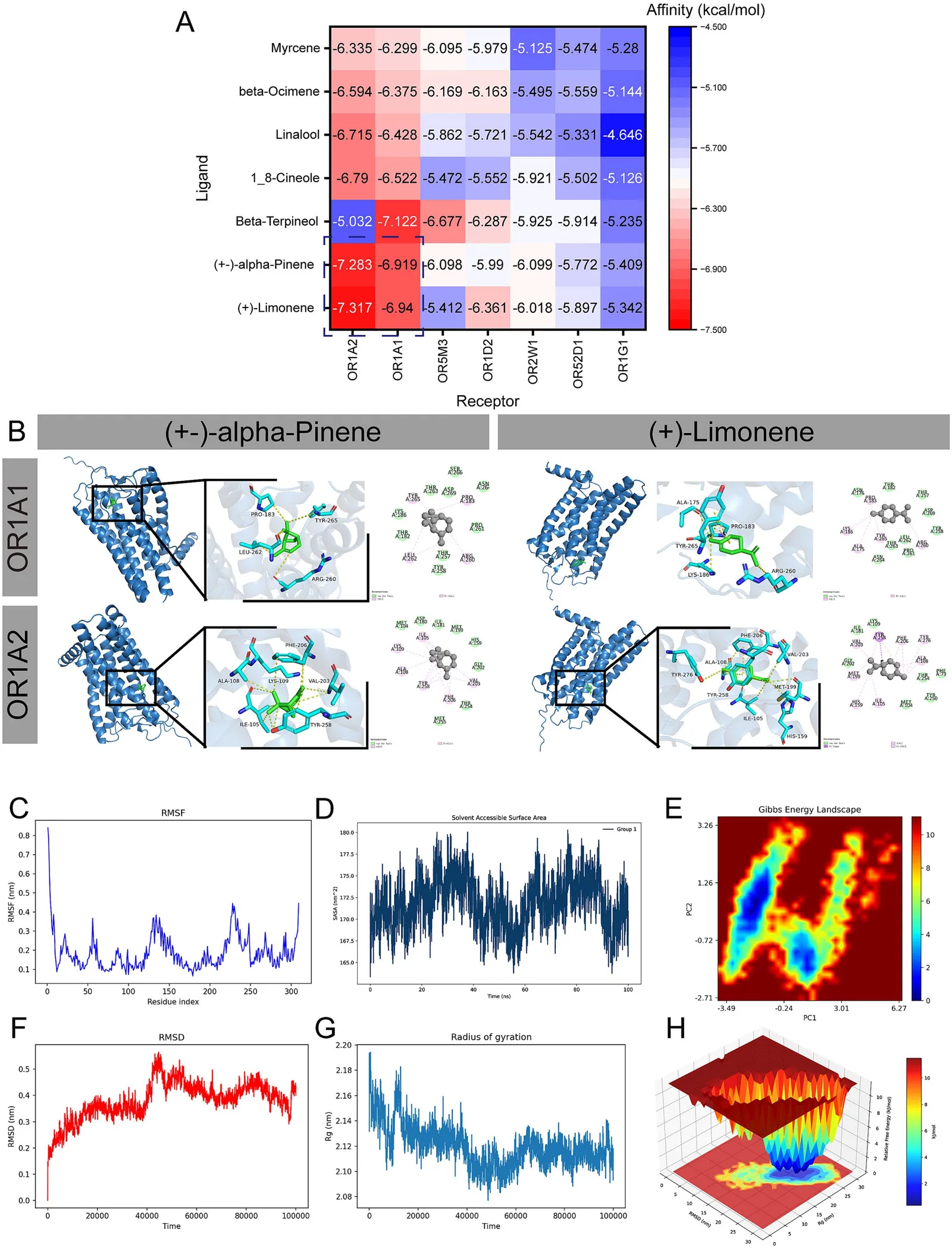
Molecular docking and molecular dynamics simulation revealing the interaction between key aroma compounds and olfactory receptors. (A) Heatmap of binding energies between seven major aroma compounds and seven olfactory receptors. (B) Detailed docking poses of α-pinene and limonene within the binding pockets of OR1A1 and OR1A2, respectively. (C) Root-mean-square fluctuation (RMSF) of the receptor residues in the (+)-limonene–OR1A2 complex during simulation. (D) Solvent accessible surface area (SASA) of the (+)-limonene–OR1A2 complex. (E) Two-dimensional Gibbs free energy landscape (2D FEL) of the complex. (F) Root-mean-square deviation (RMSD) of the complex over the 100 ns simulation. (G) Radius of gyration (Rg) of the complex. (H) Three-dimensional Gibbs free energy landscape (3D FEL) of the complex.

OR1A2, one of the most broadly tuned members of the human olfactory receptor family, has been demonstrated to be activated by various terpenoid compounds (Xiao et al., 2024). The present study further provides molecular dynamics-based evidence for the conformational stability of the complex on the nanosecond timescale. The narrow fluctuation of Rg (Fig. 5G) and the relatively rigid scaffold indicated by the RMSF analysis suggest that the (+)-limonene and OR1A2 complex maintained a stable conformation throughout the simulation rather than exhibiting substantial structural dissociation. These findings provide a potential molecular-level explanation for the prominent contribution of limonene to the overall aroma profile of Sichuan pepper and offer a potential receptor target for further development of a molecular evaluation system for Sichuan pepper aroma quality.

### 3.8 Changes in the contents of non-volatile constituents

The changes in the contents of non-volatile constituents are shown in Fig. 6A, and the corresponding HPLC chromatograms are presented in Fig. 6B. The moisture content decreased progressively with increasing stir-frying time (Fig. S3), indicating substantial water loss during thermal processing. Therefore, the contents of non-volatile constituents were expressed on a dry-weight basis according to the measured moisture content at each corresponding stir-frying time point, thereby minimizing the potential influence of dehydration on apparent concentration changes. In the raw sample, the total flavonoid content, expressed as rutin equivalents (RE), and total polyphenol content, expressed as gallic acid equivalents (GAE), were 243.43 and 205.38 mg/g DW, respectively. Total flavonoids increased progressively during the early-to-middle stages of stir-frying, reaching a maximum of 297.96 mg/g DW at 5 min, and subsequently decreased to 254.30 mg/g DW at 10 min. In contrast, total polyphenols decreased continuously from 205.38 mg/g DW in the raw sample to 160.38 mg/g DW at 5 min and 150.23 mg/g DW at 10 min. The increase in total flavonoids may be attributable to enhanced extractability and heat-induced transformation, whereas the continuous decline of total polyphenols may reflect their thermal sensitivity and heat-induced degradation. Hydroxy-β-sanshool and hydroxy-α-sanshool, two major numbing-related constituents, both reached their maximum levels at 5 min, at 7.47 and 7.58 mg/g, respectively, and subsequently decreased to 5.23 and 5.16 mg/g at 10 min. Xanthoxylin increased during the early-to-middle stages, reached a maximum of 54.02 mg/g at 6 min, and then declined to 25.89 mg/g at 10 min. Rutin also increased markedly, reaching a maximum of 8.05 mg/g at 5 min, followed by a gradual decrease during prolonged heating. These dynamic changes indicate that individual non-volatile constituents responded differently to thermal processing, with transient increases potentially reflecting changes in extractability, release, or thermal transformation rather than dehydration alone. Cluster analysis and PCA (Fig. 6C, D) separated the raw sample from the stir-fried samples and further distinguished the 9–10 min samples from the moderate stir-frying group represented by the 1–8 min samples. Because the total flavonoid and total polyphenol assays are based on non-specific colorimetric reactions, their values should be interpreted as equivalent-based estimates rather than absolute concentrations of flavonoids or polyphenols. Notably, the two major sanshool compounds reached their maximum levels at 5 min, suggesting that this stage represents an important compositional transition before the more pronounced decline in numbing-related constituents during prolonged heating, consistent with the reported thermal instability of sanshool compounds (Duan et al., 2024).

**Fig. 6:**
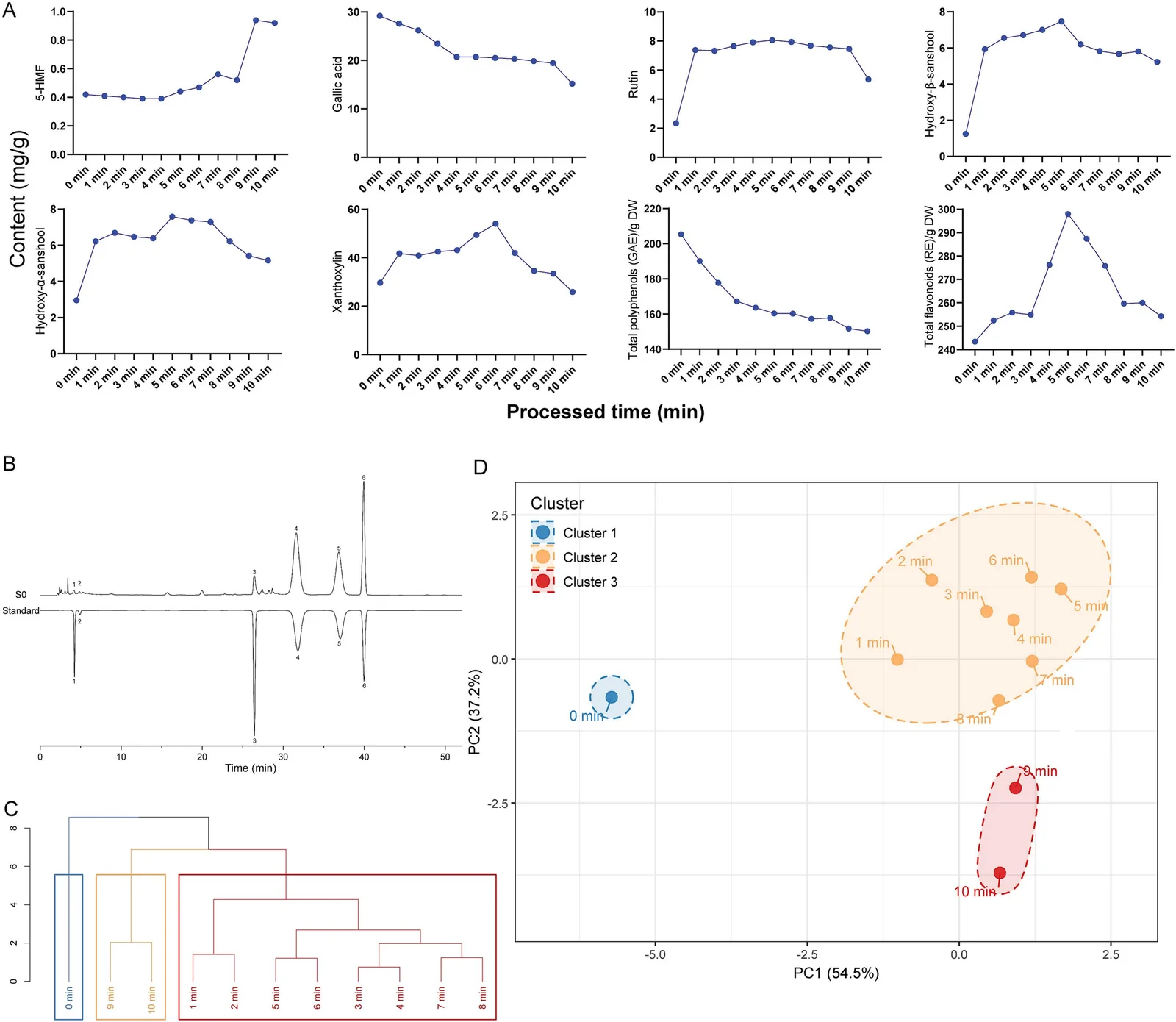
Dynamics of non-volatile constituents during stir-frying. (A) Changes in the contents of total flavonoids, total polyphenols, hydroxy-α-sanshool, hydroxy-β-sanshool, xanthoxylin, gallic acid, rutin, and 5-HMF. (B) Representative HPLC–UV chromatograms of samples at selected time points. (C) Hierarchical cluster analysis based on non-volatile components. (D) PCA score plot classifying the samples into under-stir-fried, moderate, and over-stir-fried groups.

### 3.9 Antioxidant activity analysis

The changes in antioxidant activity are illustrated in Fig. 7A-D. All four indices exhibited an initial increase followed by a decrease, with their peak values observed at 5 min of stir-frying. The hydroxyl radical scavenging rate rose from 33.80% in the raw sample to 65.73%, nearly doubling; the DPPH radical scavenging rate increased to 44.13%; the superoxide anion scavenging activity reached 315.00 U/L, representing an approximately 28.6% increase relative to the raw sample; and the ABTS radical cation scavenging capacity also increased to 0.881. After 5 min, all indices gradually declined, and by 10 min they were significantly lower than those of the unstir-fried sample, demonstrating that prolonged exposure to high temperature may substantially reduce antioxidant capacity. The enhancement of antioxidant activity during moderate stir-frying may be attributable to the accumulation of Maillard reaction products and the thermal release of phenolic compounds from bound forms. The antioxidant response was temporally consistent with the compositional changes observed in the non-volatile constituents, particularly the transient accumulation of several antioxidant-related components during the earlier stages and their subsequent decline during prolonged heating. Sustained high-temperature exposure may therefore have contributed to the decline in antioxidant capacity at later stages. These findings further support 5 min as a reference processing time for quality preservation. This interpretation is consistent with the compositional changes described in Section 3.8, in which several non-volatile constituents increased during the early-to-middle stages but declined with prolonged heating. Thus, the antioxidant peak at 5 min likely reflects a temporary balance between the release or formation of antioxidant-active substances and the onset of thermal degradation.

**Fig. 7:**
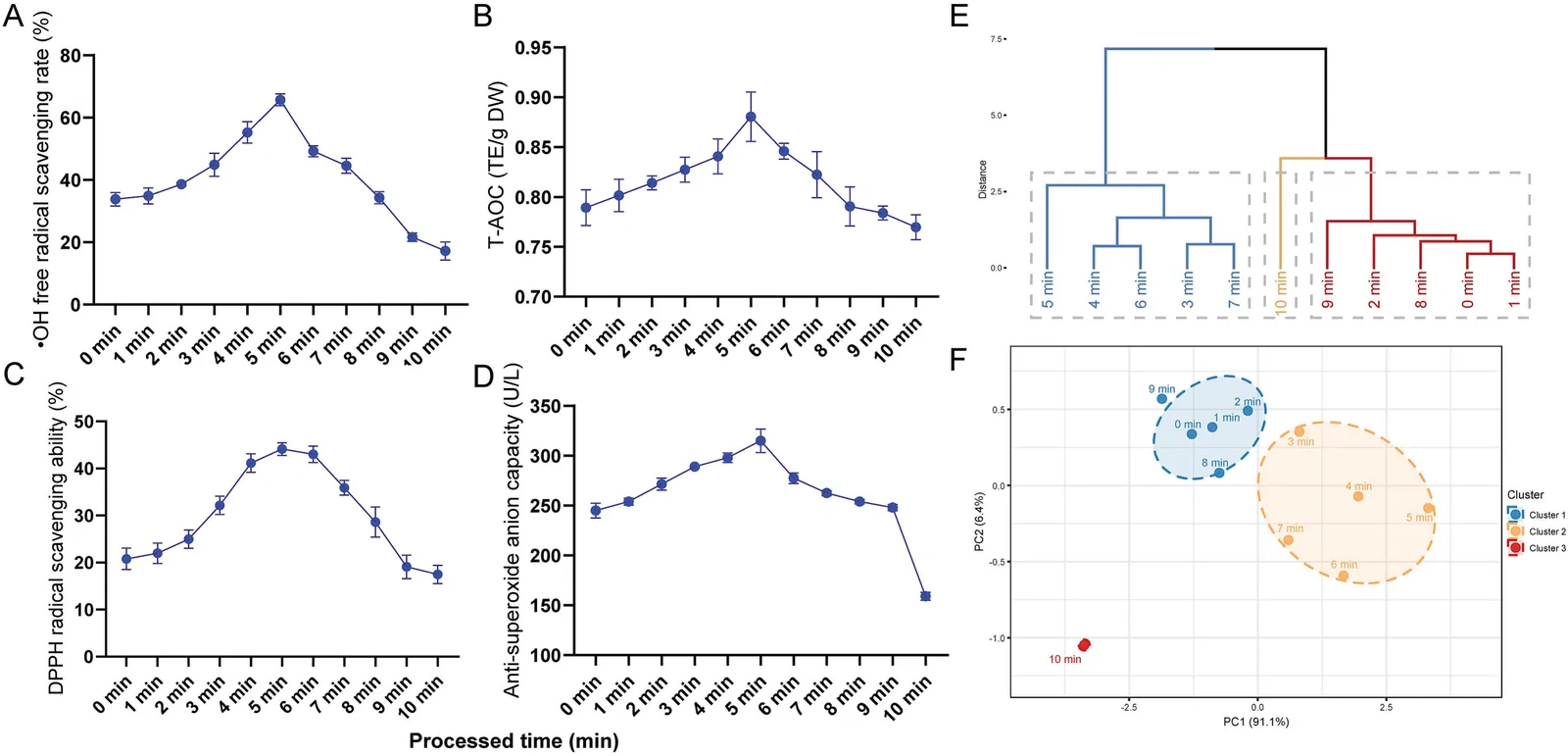
Variations in antioxidant activity during stir-frying. (A) Hydroxyl radical (·OH) scavenging rate. (B) ABTS radical cation scavenging capacity (expressed as TEAC, mmol TE/g DW). (C) DPPH radical scavenging rate. (D) Superoxide anion radical scavenging activity. (E) Hierarchical cluster analysis dendrogram based on antioxidant activity parameters. (F) Principal component analysis (PCA) score plot of samples categorized by stir-frying time.

### 3.10 Correlation analysis among chemical composition, aroma, and color

To elucidate the synergistic changes among chemical composition, aroma, color, and sensory attributes during the stir-frying of Sichuan pepper, key quality indicators obtained from instrumental, chemical, antioxidant, and sensory analyses were systematically examined. Cluster analysis (Fig. 8A) grouped the samples at different stir-frying times into three major clusters: 0 min and 2 min formed a cluster corresponding to the early processing stage; 4, 5, 7, and 8 min clustered together as the middle processing stage; and 1, 3, 6, 9, and 10 min formed another cluster representing a later processing stage. This classification, together with the observed changes in color, sensor responses, volatile compounds, and chemical constituents, indicated that quality transformation during stir-frying was not strictly linear. The distribution of certain time points (e.g., 2 min in the early-stage cluster and 1 min and 3 min in the later-stage cluster) further suggested fluctuations in the early processing stage, which may be related to sample heterogeneity and the nonlinear nature of compositional changes. Principal component analysis (PCA, Fig. 8B) further corroborated the above clustering trend, with the first two principal components (PC1 and PC2) cumulatively explaining 82.1% of the total variance. Samples of different stir-frying times were distributed orderly along PC1, with 0 min and 2 min located at opposite ends of the score plot, while samples from the middle stage were relatively concentrated in the central region, indicating that stir-frying time exerted a major influence on the overall measured quality characteristics of Sichuan pepper.

**Fig. 8:**
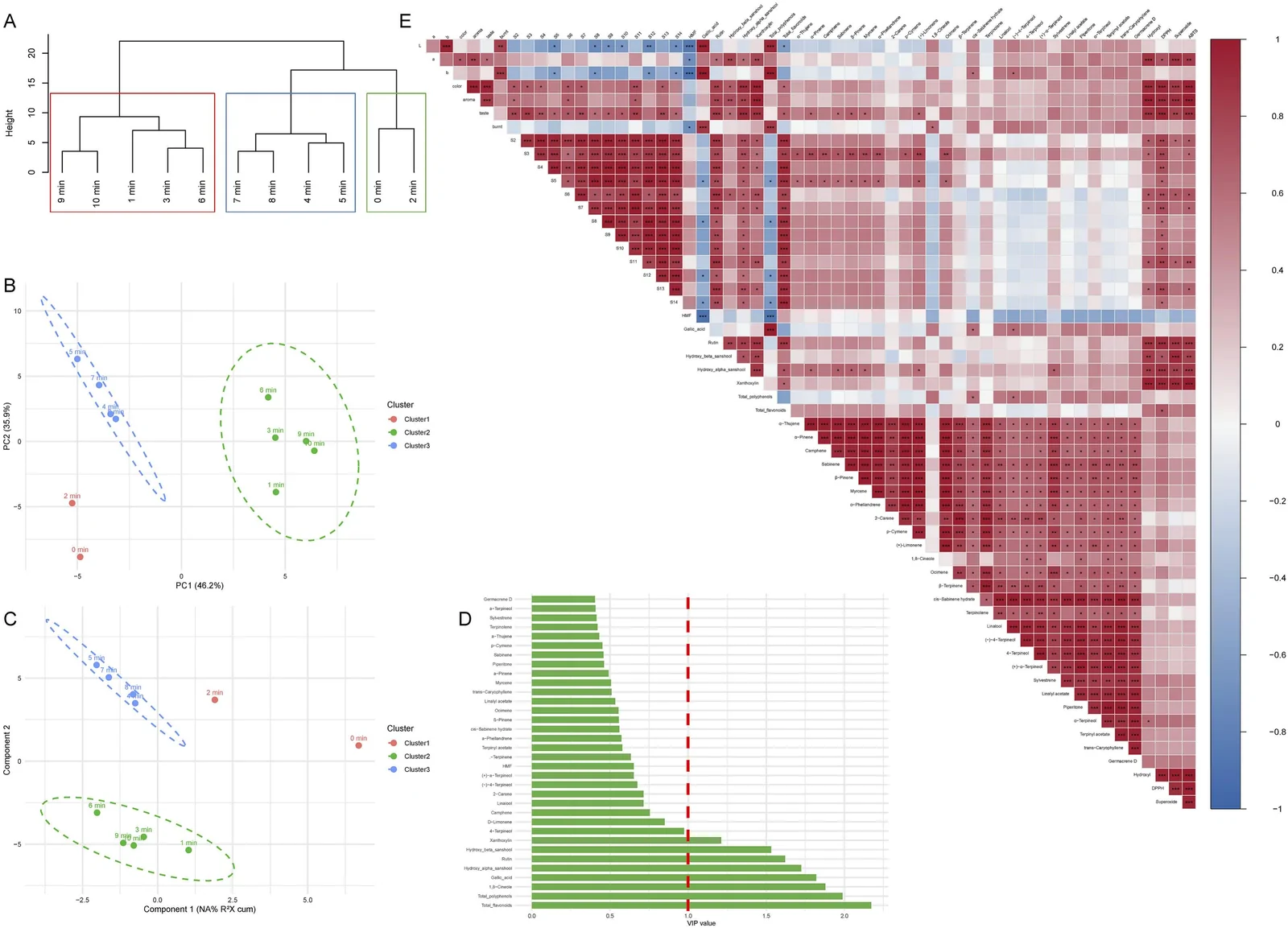
Multivariate correlation analysis among chemical composition, aroma, color, and antioxidant indicators. (A) Hierarchical cluster analysis of samples based on 22 quality-related parameters. (B) PCA score plot with PC1 and PC2 explaining 82.1% of the total variance. (C) Partial least squares regression (PLSR) correlation loading plot illustrating relationships between predictor variables (chemical constituents) and response variables related to color and aroma. (D) Variable importance in projection (VIP) scores, highlighting seven key contributors with VIP > 1. (E) Spearman correlation heatmap showing pairwise correlations among color attributes, bioactive compounds, numbing-taste substances, and antioxidant indices (*P < 0.05, **P < 0.01, ***P < 0.001).

Partial least squares regression (PLSR, Fig. 8C) was performed using color parameters (L*, a*, b*) and ROAVs of key aroma compounds as response variables, and the contents of volatile and non-volatile constituents as predictor variables. Variable importance in projection (VIP) analysis (Fig. 8D) revealed that seven components, including total flavonoids, total polyphenols, eucalyptol (1,8-cineole), and gallic acid, were important contributors to the observed “color–aroma” changes, with VIP values exceeding 1.0. These components maintained relatively high levels during the early-to-middle stage of stir-frying (0–5 min) and declined synchronously thereafter, whereas the VIP values of charring products increased markedly in the late stage of stir-frying. These results suggest that both volatile and non-volatile constituents contributed to the coordinated changes in color and aroma, rather than a single component determining the overall quality changes. The VIP values of total flavonoids and total polyphenols exceeding 1 highlight the central importance of phenolic compounds in the association between color browning and bioactivity attenuation.

Spearman correlation analysis (Fig. 8E) further delineated the relationships among the indicators, including color attributes, bioactive compounds, numbing-taste substances, and antioxidant indices. The L* value was significantly correlated with total flavonoids, total polyphenols, and gallic acid, showing a negative correlation with total flavonoids (*r* = −0.61, *P* < 0.05) but positive correlations with total polyphenols (*r* = 0.95, *P* < 0.001) and gallic acid (*r* = 0.96, *P* < 0.001), indicating that changes in lightness were closely associated with variations in these chemical constituents. In contrast, L* was significantly negatively correlated with 5-HMF (*r* = −0.86, *P* < 0.001), consistent with the progressive accumulation of Maillard reaction products during prolonged stir-frying. The a* value was significantly positively correlated with numbing-related components**,** including hydroxy-α-sanshool, xanthoxylin, and hydroxy-β-sanshool, and showed positive correlations with hydroxy-α-sanshool (*r* = 0.62, *P* < 0.05), hydroxy-β-sanshool (*r* = 0.80, *P* < 0.01), and xanthoxylin (*r* = 0.78, *P* < 0.01), as well as with hydroxyl radical scavenging activity (*r* = 0.88, *P* < 0.001), DPPH scavenging activity (*r* = 0.73, *P* < 0.05), superoxide anion scavenging activity (*r* = 0.89, *P* < 0.001), and ABTS capacity (*r* = 0.85, *P* < 0.01). Together, these correlations indicate that color changes were associated with changes in bioactive and numbing-related constituents as well as antioxidant status. Sensory attributes were also closely associated with these chemical and instrumental indicators. Sensory color correlated strongly with aroma (*r* = 0.96, *P* < 0.001) and taste (*r* = 0.95, *P* < 0.001), while sensory aroma correlated with xanthoxylin (*r* = 0.97, *P* < 0.001) and ABTS capacity (*r* = 0.98, *P* < 0.001). Sensory taste was strongly correlated with hydroxy-α-sanshool (*r* = 0.92, *P* < 0.001), linking compositional changes with sensory perception. Overall, the integrated analyses demonstrated coordinated transformations in chemical composition, aroma-related characteristics, color, and sensory perception during stir-frying. Considering the combined changes in these quality-related indicators, 4–5 min was identified as a reference processing window for standardized production.

## 4 Conclusion

This study systematically characterized the dynamic changes and interrelationships among chemical composition, aroma, and color during the stir-frying of Sichuan pepper. Stir-frying time markedly affected all three attributes, and the quality transformation showed nonlinear characteristics. The L* value was significantly positively correlated with total flavonoids and total polyphenols, supporting its potential use as a visual indicator of quality changes, while a* was positively correlated with numbing-related constituents and antioxidant activity. PLSR identified seven key synergistic contributors (VIP > 1) involved in the coordinated color–aroma changes. By integrating colorimetry, electronic-nose analysis, HS-GC–MS, HPLC–UV, antioxidant assays, sensory evaluation, and molecular docking and dynamics simulations, this study provides a multidimensional framework for characterizing the coordinated quality changes during stir-frying. Taken together, the coordinated changes across the measured indicators support 4–5 min as a reference processing window based on the integrated quality indicators**,** rather than defining aroma quality from any single instrumental measurement alone.

## CRediT authorship contribution statement

**Kangjie Li:** Writing – original draft, Validation, Investigation, Formal analysis, Data curation. **Jing Wang:** Investigation, Data curation. **Xiaofei Zhang:** Software, Investigation. **Qiao Zhang:** Methodology, Investigation. **Jing Sun:** Methodology, Funding acquisition. **Chongbo Zhao:** Supervision, Resources, Project administration, Funding acquisition, Conceptualization.

## Funding

This work was supported by Key scientific research program of Shaanxi Provincial Department of education (24JR055), the High-level Key Discipline of Chinese Medicinal Processing in Shaanxi Province, the Key Research and Development Plan of Shaanxi Province (2024SF-ZDCYL-03-11), Shaanxi Provincial Department of science and technology (2025JC-YBMS-923, 2025JC-YBMS-913), Shaanxi Provincial Department of Science and Technology program (No. 2024SF-YBXM-483), Key Research & Development Program of Shaanxi Provincial Department of Science and Technology (2024CY-JJQ-36).

## Declaration of competing interest

The authors declare that they have no known competing financial interests or personal relationships that could have appeared to influence the work reported in this paper.

## Data Availability

Data will be made available on request.

## References

[bb0005] Abraham, M. J., Murtola, T., Schulz, R., Páll, S., Smith, J. C., Hess, B., & Lindahl, E. (2015). GROMACS: High performance molecular simulations through multi-level parallelism from laptops to supercomputers. SoftwareX, 1–2, 19–25. doi:10.1016/j.softx.2015.06.001.

[bb0010] Abramson J., Adler J., Dunger J., Evans R., Green T., Pritzel A., Jumper J.M. (2024). Accurate structure prediction of biomolecular interactions with AlphaFold 3. Nature.

[bb0015] Alim A., Huang C., Zhao X., Sarengaowa, Zhang R., Zhang J., Zhang X., Jin Y., Wu G., Hu W. (2025). GC-IMS/GC-MS/E-nose and molecular simulation analysis of the effects of aging duration on the aroma of Citri Reticulatae Pericarpium and its associated antidepressant effects. Journal of Agriculture and Food Research.

[bb0020] Chen K., Zhang M., Mujumdar A.S., Deng D. (2024). The control effect and mechanism of antioxidants on the flavor deterioration of Sichuan pepper essential oil during thermal processing. Food Bioscience.

[bb0025] Chinese Pharmacopoeia Commission (2025).

[bb0030] Dai J.-W., Zeng Q., Du Y.-Q., Liu Y.-W., Xiao H.-W., Qin W., Li Y.-L. (2026). Comparison and modeling of different drying technologies for *Zanthoxylum bungeanum* maxim.: Changes in drying kinetics, color, dehiscence rate, volatile oil content and amide content. Foods.

[bb0035] Ding Y., Cheng R., Li Y., Jiang D., Zhao H., Wu X., Shu Y., Lu T., Jin C., Wu D., Zhang W. (2025). Effects of stir-frying on chemical profile, sensory quality and antioxidant activity of Chrysanthemi Flos: A metabolomics and sensory study. Food Research International.

[bb0040] Dong T., Wang S., Qi N., Sun J., Chen H., Wang S., Sun B. (2024). Unraveling the influence of boiling time on aroma generation in Huajiao (*Zanthoxylum bungeanum* maxim.) water during boiling through molecular sensory science. Food Chemistry*:* X.

[bb0045] Duan P., Feng X., Peng X., Wang L., Wang H., Kan J. (2024). Degradation and transformation mechanisms of pungent substances in huajiao (Zanthoxylum bungeanum) oil during storage: Induced by ultraviolet irradiation. Food Chemistry.

[bb0050] Fei X., Ma L., Wei A., Shi Q. (2025). Regional quality differences in Zanthoxylum bungeanum using sensor-based analysis and climate factors. Food Chemistry.

[bb0055] Feng J., Zhang B., Zhang H., Wu Z., Li M., Wang D., Wang C. (2024). Combining with E-nose, GC-MS, GC-IMS and chemometrics to explore volatile characteristics during the different stages of Zanthoxylum bungeanum maxim fruits. Food Research International.

[bb0060] Gao L., Shi B., Zhao L., Wang H., Xiang Y., Zhong K. (2024). Aroma characteristics of Green Huajiao in Sichuan and Chongqing area using sensory analysis combined with GC-MS. Foods.

[bb0065] Gu J., Zhan P., Tian H., Wang P., He W., Geng J., Zhang R. (2025). Study on the aroma formation pathways in huajiao (Zanthoxylum bungeanum M.) oil during the frying process: Instrumental, sensory, and correlation analysis. Journal of Food Composition and Analysis.

[bb0070] He L., Yi Y., Jia H., Xu C., Qiao M., Cai X., Peng H. (2025). Impact of harvest periods on the physicochemical and flavour characteristics of Sichuan pepper (*Zanthoxylum bungeanum* maxim). Foods.

[bb0075] Ivane N.M.A., Haruna S.A., Zekrumah M., Roméo Elysé F.K., Hassan M.O., Hashim S.B.H., Zhang D. (2022). Composition, mechanisms of tingling paresthesia, and health benefits of Sichuan pepper: A review of recent progress. Trends in Food Science & Technology.

[bb0080] Khan M., Liu H., Wang J., Sun B. (2020). Inhibitory effect of phenolic compounds and plant extracts on the formation of advance glycation end products: A comprehensive review. Food Research International.

[bb0085] Li J., Hou X., Jiang L., Xia D., Chen A., Li S., Li Q., Gu X., Mo X., Zhang Z. (2022). Optimization and characterization of Sichuan pepper (Zanthoxylum bungeanum maxim) resin microcapsule encapsulated with β-cyclodextrin. LWT.

[bb0090] Li Z., Jeon J., Kwon S.Y., Huang R., Baek H.-H. (2019). Characterization and evaluation of changes in the aroma-active components in Szechuan pepper (Zanthoxylum bungeanum maxim) under different cooking temperatures using gas chromatography-olfactometry. Chemosensory Perception.

[bb0095] Liu R., Qi N., Sun J., Chen H., Zhang N., Sun B. (2022). Effects of frying conditions on volatile composition and odor characteristics of fried pepper (*Zanthoxylum bungeanum* maxim.) oil. Foods.

[bb0100] Ma Y., Li X., Hou L.-X., Wei A.-Z. (2019). Extraction solvent affects the antioxidant, antimicrobial, cholinesterase and HepG2 human hepatocellular carcinoma cell inhibitory activities of Zanthoxylum bungeanum pericarps and the major chemical components. Industrial Crops and Products.

[bb0105] Mahanta B.P., Bora P.K., Kemprai P., Borah G., Lal M., Haldar S. (2021). Thermolabile essential oils, aromas and flavours: Degradation pathways, effect of thermal processing and alteration of sensory quality. Food Research International.

[bb0110] Qin H., Fang S., Xu H., Jiang S., Bu J., Long H., Wu G., Yang L., Jin G., Wu Y., Mu D. (2026). Integrating E-nose, E-tongue, and GC–MS analysis reveals the volatile profile and flavor characteristics of different huajiao cultivars. Food Chemistry: X.

[bb0115] Qiu J., Gong Y., Lu S., Xv J., Zhai Y., Gong W., Hui W., Wang J. (2025). Seasonal changes of volatile compounds and odor evaluation of *Zanthoxylum bungeanum* ‘Hanyuan’ fruit during the development. BMC Plant Biology.

[bb0120] Song Y., Jia Z., Yan L., Liu Y., Cui Z., Wang Y., Zhang C. (2025). Exploring the material basis and formation pathways of the burnt aroma during the stir-frying process of Gardeniae fructus using Sensomics and chemical components approach. Food Chemistry: X.

[bb0125] Su K., Sun S., Chen R., Ren M., Wang Y., Bi S., Liu Y. (2025). Identification of key aroma-active compounds in five types of Zanthoxylum bungeanum maxim. Essential oils using a molecular sensory approach. Journal of Food Composition and Analysis.

[bb0130] Wang D., Chu F., Zhang J., Wei H. (2026). Evaluating the reliability of epicarp chromaticity as an independent indicator of maturity state during harvesting time for sweet cherry. LWT.

[bb0135] Wu H., Chen Y., Guo Y., Liang Q., Liu L., Wen H., Wu C. (2026). Investigation of the degradation patterns and mechanisms of amide compounds in Zanthoxylum oil under temperature influence. Industrial Crops and Products.

[bb0140] Xiao Z., Li Q., Niu Y., She Y., Sun Z., Zhang J., Wang Z., Zhou R., Zhu J. (2024). Mechanism of the interaction between olfactory receptors and characteristic aroma compounds in sweet orange juice. LWT.

[bb0145] Yang N., Jin Y. (2024). Novel electromagnetic heat hydrodistillation for extraction of essential oil from tangerine peel. Foods.

[bb0150] Zhang K., Wang S., Zhang H., Ming J., Long W., Fu H. (2026). Revealing the chemical differences and their application on geographical origin and aging year identification of Artemisia Argyi folium based on GC–MS and UHPLC-HRMS untargeted metabolomics. Food Research International.

[bb0155] Zhang W., Li M., Chai Y., Huang Y., Jin Y., Ma J., Jiang Y., Tang Y. (2025). Cutting morphology and steaming duration impact physicochemical quality, bioactive retention, and maillard reaction products in purple-fleshed sweet potatoes. Food Chemistry.

[bb0160] Zhao M., Li T., Yang F., Cui X., Zou T., Song H., Liu Y. (2022). Characterization of key aroma-active compounds in Hanyuan Zanthoxylum bungeanum by GC-O-MS and switchable GC × GC-O-MS. Food Chemistry.

[bb0165] Zhu X., Wu D., Zhao L., Wen C., Yong C., Kan Q. (2025). Unveiling the flavor and quality variations in dried Zanthoxylum bungeanum maxim from China's diverse regions. Food Chemistry: X.

[bb0170] Zuo H., Si X., Li P., Li J., Chen Z., Li P., Chen C., Liu Z., Zhao J. (2023). Dynamic change of tea (Camellia sinensis) leaf cuticular wax in white tea processing for contribution to tea flavor formation. Food Research International.

