# Supplementary material for: Synergistic changes and interrelationships among chemical composition, aroma, and color during the stir-frying of Sichuan pepper (Zanthoxylum bungeanum maxim.)
Source: Food Chem X. 2026 Oct 16;40:104644. doi: 10.1016/j.fochx.2026.104644 (PMC13697283; doi:10.1016/j.fochx.2026.104644)
Supplement: Supplementary file 1 [file mmc1.docx]

**Fig. S1** Comparison of temperature–time profiles of Sichuan pepper during stir-frying using an electric ceramic cooktop and a traditional open-flame method.

**Fig. S2** Higher-magnification SEM images (2000×) of Sichuan pepper at different stir-frying times, showing detailed changes in surface micropore formation, oil release, and tissue collapse.

**Fig. S3** Changes in moisture content of Sichuan pepper during stir-frying.

**Table S1.** Odor thresholds, media, and references used for ROAV calculation.

| No. | Compound | Odor threshold (T) | Unit | Medium | Reference |
| --- | --- | --- | --- | --- | --- |
| 1 | Alpha-Thujene | 0.001 | mg/kg | water | (Huang et al., 2022) |
| 2 | Alpha-Pinene | 0.274 | mg/kg | oil | (L.J. van Gemert, 2011) |
| 3 | Camphene | 0.015 | mg/kg | water | (Xie et al., 2026) |
| 4 | Sabinene | 0.98 | mg/kg | water | (L.J. van Gemert, 2011) |
| 5 | beta-Pinene | 0.14 | mg/kg | water | (L.J. van Gemert, 2011) |
| 6 | Myrcene | 0.046 | mg/kg | water | (L.J. van Gemert, 2011) |
| 7 | Alpha-Phellandrene | 0.04 | mg/kg | water | (L.J. van Gemert, 2011) |
| 8 | (+)-2-Carene | 0.21 | mg/kg | water | (Pu et al., 2017) |
| 9 | P-Cymene | 0.0133 | mg/kg | water | (Xie et al., 2026) |
| 10 | D-Limonene | 0.034 | mg/kg | water | (L.J. van Gemert, 2011) |
| 11 | Eucalyptol | 0.023 | mg/kg | water | (L.J. van Gemert, 2011) |
| 12 | Ocimene | 0.034 | mg/kg | water | (L.J. van Gemert, 2011) |
| 13 | Gamma -Terpinene | 1 | mg/kg | water | (L.J. van Gemert, 2011) |
| 14 | 4-Thujanol | 0.03 | mg/kg | water | (L.J. van Gemert, 2011) |
| 15 | Terpinolene | 0.2 | mg/kg | water | (L.J. van Gemert, 2011) |
| 16 | Linalool | 0.006 | mg/kg | water | (L.J. van Gemert, 2011) |
| 17 | beta-Terpineol | 0.003 | mg/kg | water | (Li et al., 2025) |
| 18 | Terpinen-4-ol | 1.2 | mg/kg | water | (Kang et al., 2026) |
| 19 | (+)-alpha-Terpineol | 0.3 | mg/kg | water | (Kang et al., 2026) |
| 20 | Sylvestrene | 0.0024 | mg/kg | water | (L.J. van Gemert, 2011) |
| 21 | Linalyl acetate | 1 | mg/kg | water | (L.J. van Gemert, 2011) |
| 22 | Piperitone | 0.68 | mg/kg | water | (L.J. van Gemert, 2011) |
| 23 | Alpha-Terpineol | 0.23 | mg/kg | water | (L.J. van Gemert, 2011) |
| 24 | Alpha-Terpinyl | 2.5 | mg/kg | water | (L.J. van Gemert, 2011) |
| 25 | Beta-Caryophyllen | 0.064 | mg/kg | water | (L.J. van Gemert, 2011) |
| 26 | Germacrene D | 1.2 | mg/kg | water | (Li et al., 2025) |

**Table S2.** The linear regression equations, linearity range and correlation coefficients

| No | Compounds name | Regression equation | Correlation  coefficient | linearity range (mg/mL) |
| --- | --- | --- | --- | --- |
| 1 | HMF | y = 5,156,050.0237 x - 177,215.7032 | R² = 0.9959 | 0.0529-1.0581 |
| 2 | Gallic acid | y = 95,350.9570 x + 15,388.1945 | R² = 0.9989 | 0.3516-7.0323 |
| 3 | Rutin | y = 2,709,254.2270 x + 94,865.2421 | R² = 0.9998 | 0.3194-6.3871 |
| 4 | Hydroxy-β-sanshool | y = 7,455,713.5284 x + 729,597.3646 | R² = 0.9952 | 0.1145-2.2903 |
| 5 | Hydroxy-α-sanshool | y = 1,911,461.4122 x + 281,321.7089 | R² = 0.9989 | 0.3194-6.3871 |
| 6 | Xanthoxylin | y = 1,963,062.3184 x + 119,852.9078 | R² = 0.9996 | 0.3016-6.0323 |

**Table S3** The results of the precision, stability, repeatability and recovery (n=6)

| Compounds | Precision | Stability | Repeatability | Average recovery rate | Recovery |
| --- | --- | --- | --- | --- | --- |
|  | RSD (%) | RSD (%) | RSD (%) | % | RSD (%) |
| HMF | 0.68 | 0.65 | 1.27 | 96.17 | 0.73 |
| Gallic acid | 0.78 | 0.97 | 1.35 | 97.12 | 1.06 |
| Rutin | 1.20 | 1.43 | 0.89 | 96.64 | 0.89 |
| Hydroxy-β-sanshool | 1.06 | 1.27 | 1.22 | 97.63 | 0.61 |
| Hydroxy-α-sanshool | 1.06 | 1.43 | 1.34 | 97.96 | 1.24 |
| Xanthoxylin | 0.97 | 1.25 | 1.00 | 98.43 | 0.93 |

**References**

Huang, ganghua, Li, Y., & Deng, X. (2022). Analysis of Aroma Compounds of Yunnan White Tea byFour Drying Methods. *Science and Technology of Food Industry*, *43*(18), 283–292.

Kang, J., Wen, H., Wu, Q., & AO, Y. (2026). Comparative Analysis of Volatile Compounds in Seven Citrus junos Germplasms. *Acta Horticulturae Sinica*, *53*(1), 25–40.

Li, C., Chen, W., & Zheng, H. (2025). Comprehensive Quality Evaluation of Thick-Skinned Muskmelons Based on Nutritional Composition and Aroma Analysis. *Food Science*, *46*(13), 253–265.

L.J. van Gemert. (2011). *Odour Thresholds: Compilations of Odour Threshold Values in Air, Water and Other Media* (2nd ed.). Oliemans Punter & Partners BV.

Pu, F., Sun, W., Che, Z., & Liu, Y. (2017). Analysis of Volatile Aroma Components in Hanyuan Green and Red Peppers by Steam Distillation and GC-MS. *China Condiment*, *42*(1), 23–27.

Xie, Q., Luo, J., Liang, L., Su, W., Lyu, M., Peng, C., Li, B., Wang, W., & Yuan, H. (2026). Effects of Different Drying Methods on the Flavor Characteristics and Chemical Profile of Forsythia suspensa Flowers Using Electronic Sensors and Mass Spectrometry. *Foods*, *15*(10). https://doi.org/10.3390/foods15101757
